# The Identification of Prognostic Factors and Survival Statistics of Conventional Central Chondrosarcoma

**DOI:** 10.1155/2015/623746

**Published:** 2015-11-08

**Authors:** Sjoerd P. F. T. Nota, Yvonne Braun, Joseph H. Schwab, C. Niek van Dijk, Jos A. M. Bramer

**Affiliations:** ^1^Orthopaedic Oncology Service, Massachusetts General Hospital, Harvard Medical School, Boston, MA 02114, USA; ^2^Department of Orthopaedic Surgery, Academic Medical Center, 1105 AZ Amsterdam, Netherlands

## Abstract

*Introduction*. Chondrosarcomas are malignant bone tumors that are characterized by the production of chondroid tissue. Since radiation therapy and chemotherapy have limited effect on chondrosarcoma, treatment of most patients depends on surgical resection. We conducted this study to identify independent predictive factors and survival characteristics for conventional central chondrosarcoma and dedifferentiated central chondrosarcoma. *Methods*. A systematic literature review was performed in September 2014 using the Pubmed, Embase, and Cochrane databases. Subsequent to a beforehand-composed selection procedure we included 13 studies, comprising a total of 1114 patients. *Results*. The prognosis of central chondrosarcoma is generally good for the histologically low-grade tumors. Prognosis for the high-grade chondrosarcoma and the dedifferentiated chondrosarcoma is poor with lower survival rates. Poor prognostic factors in conventional chondrosarcoma for overall survival are high-grade tumors and axial/pelvic tumor location. In dedifferentiated chondrosarcoma the percentage of dedifferentiated component has significant influence on disease-free survival. *Conclusion*. Despite the fact that there are multiple prognostic factors identified, as shown in this study, there is a need for prospective and comparative studies. The resulting knowledge about prognostic factors and survival can give direction in the development of better therapies. This could eventually lead to an evidence-based foundation for treating chondrosarcoma patients.

## 1. Introduction

Chondrosarcomas are malignant bone tumors that can be characterized by the production of chondroid tissue [1]. This heterogeneous group of tumors occupy about a quarter of all the primary malignant osseous neoplasms of the bone [2]. Chondrosarcomas are the most common occurring primary sarcoma of the bone after osteosarcoma [2, 3]. The clinical behavior and prognosis of these tumors depend on many variables of which tumor grade is one of the most important; high-grade tumors have a worse prognosis compared to low-grade tumors [4, 5]. This poor prognosis can partially be explained by the high tendency to metastasize. About three-quarters of all chondrosarcomas consist of conventional central chondrosarcoma. These central chondrosarcomas have the outgrowth of the sarcomatous tumor in the intramedullary cavity in common. The central chondrosarcoma's anatomical counterpart is the peripheral chondrosarcoma. These specific chondrosarcomas develop from a preexisting osteochondroma and are situated on the outside of the cortex of the bone. The peripheral chondrosarcoma tumors have a better prognosis when compared to the central chondrosarcoma and tend to affect younger patients [6].

Radiation therapy and chemotherapy have limited to arguably no effect on conventional chondrosarcoma [7, 8]. There are rarer chondrosarcoma subtypes that are more responsive to chemotherapy and/or radiation therapy [9]. The vast majority of chondrosarcoma patients solely depended on the surgical treatment by tumor resection. Chemotherapy might have a role in dedifferentiated chondrosarcoma [10, 11] although the positive effect is not consistently reported in literature [11–13].

Identification of prognostic factors and knowledge about survival are important. For patients this knowledge can provide insight into their future perspective and it may provide guidance in the decision-making concerning treatment. Physicians can use the prognostic and survival information as a tool to select the optimal treatment strategy and inform patients. To direct efforts in the development of new therapeutic strategies the identification of proven prognostic factors of central chondrosarcoma is important, especially since the treatment options are limited. We conducted this systematic review with the aim of identifying independent predictive factors and survival characteristics for both conventional central chondrosarcoma and dedifferentiated central chondrosarcoma.

## 2. Materials and Methods

This systematic review was registered on PROSPERO prior to data extraction (registration number: CRD42014008961). The MOOSE checklist for meta-analysis of observational studies in epidemiology study was applied for the evaluation of meta-analysis and observational studies [14].

### 2.1. Search Strategy

We searched Pubmed, Embase, and the Cochrane database for title and abstract, without any limits on September 9, 2014, using the following search terms: ((“chondrosarcoma^*∗*^” OR “chondroid sarcoma” OR “chondroid sarcomas”) AND “prognos^*∗*^”) OR ((“chondrosarcoma^*∗*^” OR “chondroid sarcoma” OR “chondroid sarcomas”) AND “surviv^*∗*^”) resulting in a total of 2253 publications.

### 2.2. Study Selection, Data Extraction, and Critical Appraisal

Two reviewers (Sjoerd P. F. T. Nota, Yvonne Braun) independently screened all the studies' titles and abstracts and retrieved the full-text manuscripts for the articles that met our inclusion criteria. If consensus was not reached between the two reviewers, a third reviewer (Jos A. M. Bramer) was consulted. We included all articles focusing on any prognostic factors and/or survival statistics on all grades (including dedifferentiated chondrosarcoma) of primary central chondrosarcoma of the bone.

We excluded congress proceedings, letter to the editors, cohorts that were not independently identifiable, all studies published in a different language than English, and studies published before 1980. In addition we excluded case-reports and case-series with less than 10 patients. Furthermore we excluded papers reporting on surgical procedures and studies focusing solely on metastasis. Finally we excluded all papers that did not clearly distinguish between central and peripheral chondrosarcoma and reviews were excluded as well.

After applying our exclusion criteria on the title and abstract 274 papers remained for full-text screening.

The quality of the data was assessed by application of predetermined critical appraisal criteria by two independent researchers. Lack of consensus was solved again as described above. The criteria assessed were as follows: study participation, study attrition, prognostic factor measurement, outcome measurement, confounding measurement, analysis performed, population included, the time of follow-up, the level of evidence, the presence of a disclosure statement, and the presence of a baseline characteristics table (Appendix).

### 2.3. Outcome Measurements

We extracted the data of the following variables from the selected studies: author/year, type of study, mean age, sex distribution, mean duration of follow-up, primary tumors only, metastasis at presentation, grading method, tumor grade, anatomical location, overall survival, and 5- and 10-year survival per grade. In addition we registered the disease-free survival, the percentage of patients with no evidence of disease, and the percentage of patients with no evidence of disease after tumor relapse. Furthermore we looked at the percentage of patients alive with disease and dead of disease and the percentage of patients that died of a different cause. We also looked at the local recurrence rate, the time to local recurrence, metastasis rate and time to metastasis, and the use of chemo- and radiation therapy. Finally to account for the homogeneity of the treatment of the patients in the studies we also reported the status of the surgical margins of the included subjects.

### 2.4. Analysis

To prevent reporting biased results due to the high quantity of cohort studies and case-series and potential overlap of patients' population we choose to only report our results narratively and did not attempt to merge results and do additional analyses.

### 2.5. Prognostic Factors and Survival Statistics

In this review we will narratively summarize the prognostic factors and survival statistics reported in our selected studies.

### 2.6. Study Characteristic

After screening the full-text articles we included 13 studies that met our inclusion criteria for this review [10, 15–26]. The 13 studies included were based on retrospective evidence. All studies reported clearly the dates of researched period, the patient sample, and the point of the course of the disease. Nine out of the 13 studies (69%) reported a sufficient long follow-up (more than 1 year) and explained the reason of patients being lost to follow-up. Four studies (31%) did not report these factors and may therefore be subject to more selection bias (see the appendix).

### 2.7. Study Population

The 13 included studies comprised the data of 1114 patients, although population overlap is likely since multiple studies are performed in the same institution. In the studies where we could determine the age the average age of the patients ranged from 35 to 59 years and the percentage of males ranged from 42% to 79% with only 1 study reporting more females in the cohort. The mean follow-up ranged from at least more than 2 years to 13 years. The individual follow-up ranged from a minimum of 0 years to a maximum of 26 years (Table 1).

Not all studies mentioned the fact if only primary tumors and if recurrences were excluded or did included patient with such tumors (Table 2). There were a wide variety of tumor grades in the included studies. Three studies focused on central dedifferentiated chondrosarcoma and 1 study focused on grade 2 chondrosarcoma only; all other studies included patients with a variety of different tumor grades. The localizations of the tumors comprise the entire skeleton throughout the different studies (Table 2).

In 10 out of the 13 studies the surgical margin status was determined showing a wide range in the percentages of patients having wide and radical resection (Table 6).

The additional use of chemotherapy and radiotherapy is only registered in, respectively, 9 out of 13 (69%) and 6 out of 13 (46%) studies. Chemotherapy is used in 6 out of the 9 (67%) studies where chemotherapy is mentioned. Radiotherapy is used in 4 out of the 6 (67%) studies where its use is mentioned (Table 5).

## 3. Results

### 3.1. Survival: General

Overall survival ranged from 21% to 100% at the time of follow-up depending on the specific study. Five- and 10-year survival ranged from 2% to 100% and 32% to 85%, respectively (Table 3). Disease-free survival ranged from 30% to 89% and the local recurrence rate ranged from 6.2% to 35%. In the 5 studies reporting the metastasis rate the rate ranges from 0% to 38% (Table 4).

### 3.2. Survival: Grades 1, 2, and 3 and Dedifferentiated Chondrosarcoma

The reported 5-year survival for grade 1 chondrosarcoma ranged from 82% to 99%. The 10-year survival ranged from 89% to 95%. The 5-year survival for grade 2 chondrosarcoma ranged from 63% to 92%. The 10-year survival ranged from 58% to 86%. The 5-year survival for grade 3 chondrosarcoma ranged from 0% to 77%. The lowest (0%) survival was displayed in a study looking at a very small subgroup of patients treated with an intralesional resection. The 10-year survival ranged from 0% to 55%. The 5-year survival for dedifferentiated chondrosarcoma was 24% as reported in 1 study (Table 3).

### 3.3. Prognostic Factors

In 7 of the included studies prognostic factors for overall survival were reported. Cho et al. found no difference in event-free survival between curettage in combination with subsequent treatment versus standard treatment of wide excision (*p* = 0.16) in their cohort of grade 2 chondrosarcoma of the extremities [18]. Donati et al. compared survival of central with peripheral chondrosarcoma and found a difference in survival in their cohort of pelvis tumors (*p* = 0.00093) as did Gitelis et al. at 5-year (*p* < 0.001) and 10-year (*p* < 0.001) as well as in total disease-free survival (*p* < 0.005) between central and peripheral chondrosarcoma [21, 22].

Andreou et al., Angelini et al., and Staals et al. investigated multiple potential prognostic factors [15, 16, 25] as summarized in Table 7. The main significant poor prognostic factors Andreou et al. and/or Angelini et al. reported were larger tumor volume, higher grade and distant metastasis, and a worse prognosis for axial located tumors (including the pelvis) compared to in the extremity located tumors. Worst prognosis for a pathologic fracture and supportive care in comparison with multidisciplinary treatment (chemotherapy, radiotherapy, and further surgery) were reported as well. Staals et al. most prominent findings were the significant impact of Stage 3 lesions versus, respectively, Stages 2a and 2b lesions and the poor prognostic value of a higher percentage of dedifferentiated component within the tumor [25]. van Maldegem et al. show in unresectable chondrosarcoma a survival benefit for the use of chemotherapy compared to not using systemic treatment. When interpreting these results, the large heterogeneity in the treatment groups should be accounted for. In addition they show significant impact of solely unresectable disease compared to unresectable disease in combination with the presence of metastasis. Finally they showed that an age younger than 40 and grade 2 tumors have a better survival [26] (Table 7).

## 4. Discussion and Conclusions

The results of our study show that the prognosis of central chondrosarcoma is fairly good for the low histological grade tumors with a 5- and 10-year survival of over 80%. High-grade chondrosarcoma and the highly lethal dedifferentiated chondrosarcoma have a poor prognosis with lower survival rates. The main negative prognostic factors for overall survival displayed in this review are a higher tumor grade and an axial/pelvis location of the tumor for the conventional chondrosarcoma. The percentage of dedifferentiated component within dedifferentiated chondrosarcoma has significant influence on disease-free survival of these tumors.

This review should be interpreted with its limitations in mind. First of all there are only limited studies in literature that describe solely central chondrosarcoma (or where the central chondrosarcomas are identifiable). The included studies are all retrospective and, even though we used strict inclusion criteria, have a large heterogeneity between patients and treatments. The heterogeneity in histologic type of grading used to evaluate the tumors, the variability in the use of chemo- and radiotherapy, and the differences in the presence of inadequate surgical margins might all have influenced our study's main outcomes. Second limitation is the likely overlap in patient population that can be explained by the centralization of care in large institutions due to the low incidence of primary orthopaedic tumors in general populations. This might introduce a bias and might amplify the experience of a single (experienced) center. Finally there is a large heterogeneity in the outcome measures, partially explained by differences in follow-up time, which makes the direct comparison and getting a general overview of the included studies challenging. This is, for example, displayed in the wide ranges in survival statistics. The grade 3 tumors have a range of 0–77% 5-year survival. Most likely this difference is caused by comparing a small subgroup of intralesional treated tumors with the results from a highly specialized center. Also significant interobserver variability in pathologists' histologic grading is known to be present in these types of tumors [27]. This might also directly influence the reported outcomes.

Remarkably in contrast to reports in literature on chondrosarcoma [28, 29], surgical margins were not identified as independent predictor of survival in this review. However, as stated by Andreou et al. as well, in multivariable analysis Lee et al. showed only a small effect of surgical margin status on survival and Fiorenza et al. were not able to determine the effect when accounting for confounders factors [15]. Caution is needed when interpreting these conclusions and their potential consequences in practice. Relative small retrospective studies with a large heterogeneity of patients might be the cause of the inability to identify, in oncology commonly accepted, prognostic factors such as wide surgical margins.

Our study points out that there is a need for prospective and comparative studies identifying factors and treatments influencing the survival of patients suffering from central chondrosarcoma. More evidence from high quality research might eventually lead to a more evidence-based foundation of treatments while preventing abundant exposure of patients to potentially harmful therapies such as radiation and chemotherapy. Further centralization of care for patients with relatively rare diseases would be desirable from a patient's point of view but might also generate opportunities for researchers to set up prospective and comparative studies. To improve survival in central chondrosarcoma patients, the high-grade chondrosarcoma and the dedifferentiated chondrosarcoma seem to be good candidates for future studies exploring better treatments options due to their poor prognosis.

## Figures and Tables

**Table 1:** Demographic patient and study characteristics of the included studies.

Study	Study design	Patients	Mean age (range)	Male	Follow-up (range)
		(number)	(years)	(%)	(years)
Andreou et al., 2011 [15]	R	115	47 (14–79)	61%	12 (5–24)
Angelini et al., 2012 [16]	R	296	50 (13–88)	57%	7 (1.6–20)
Briccoli et al., 2002 [17]	R	14	·	·	5.8 (0–19)
Cho et al., 2011 [18]	R	32	·	72%	9.2 (2.6–19)
de Camargo et al., 2010 [19]	R	46	43 (17–79)	54%	8.3 (2.7–26)
Donati et al., 2010 [20]	R	31	35 (13–67)	42%	13 (5.5–25)
Donati et al., 2005 [21]	R	63	·	·	·
Gitelis et al., 1981 [22]	R	69	44 (14–78)	68%	>5 year
Mavrogenis et al., 2013 [23]	R	119	·	·	>2 year
Mitchell et al., 2000 [10]	R	14	57 (37–79)	79%	4.7 (1.7–7.5)^+^
Ozaki et al., 1996 [24]	R	21	51 (25–71)	67%	12 (5–22)
Staals et al., 2006 [25]	R	123^*∗*^	59 (24–83)	54%	2.8 (0–17)
van Maldegem et al., 2014 [26]	R	171	53 (17–90)	63%	·

^*∗*^110 patients with actual follow-up data, R = retrospective, and ^+^surviving patients.

**Table 2:** Oncologic patient and study characteristics of the included studies.

Study	Primary tumors only	Metastasis at presentation	Grading	Grade 1	Grade 2	Grade 3	Dediff.
Andreou et al., 2011 [15]	Yes	No	Evans et al.	56 (49%)	41 (36%)	18 (15%)	0
Angelini et al., 2012 [16]	·	·	Lichtenstein and Jaffe	87 (29%)	162 (55%)	47 (16%)	0
Briccoli et al., 2002 [17]	No	Yes	·	0	9 (64%)	4 (29%)	1 (7.1%)
Cho et al., 2011 [18]	No	No	Evans et al.	0	32 (100%)	0	0
de Camargo et al., 2010 [19]	Yes	Yes	Lichtenstein and Jaffe	23 (50%)	23 (50%)	0	0
Donati et al., 2010 [20]	No	No	Mirra et al. + Schiller et al.	31 (100%)	0	0	0
Donati et al., 2005 [21]	No	·	·	1 (1.6%)	44 (70%)	18 (29%)	0
Gitelis et al., 1981 [22]	·	·	·	9 (13%)	46 (67%)	14 (20%)	0
Mavrogenis et al., 2013 [23]	·	·	·	·	·	·	22 (18%)
Mitchell et al., 2000 [10]	·	·	·	0	0	0	14 (100%)
Ozaki et al., 1996 [24]	No	·	Evans et al.	11 (52%)	6 (29%)	4 (19%)	0
Staals et al., 2006 [25]	·	·	·	0	0	0	123 (100%)
van Maldegem et al., 2014 [26]	No	Yes	·	9	118	44	0

Study	Lower extremity	Upper extremity (incl. shoulder girdle)	Hand^*∗*^	Pelvis girdle (incl. sacrum)	Axial skeleton	Thorax	Other

Andreou et al., 2011 [15]	48 (42%)	20 (17%)	0	42 (37%)	5 (4.3%)	0	0
Angelini et al., 2012 [16]	134 (45%)	50 (17%)	4 (1.4%)	82 (28%)	10 (3.4%)	16 (5.4%)	0
Briccoli et al., 2002 [17]	0	0	0	0	0	14 (100%)	0
Cho et al., 2011 [18]	22 (69%)	7 (22%)	0	0	0	0	3 (9.4%)
de Camargo et al., 2010 [19]	25 (54%)	10 (22%)	4 (8.7%)	6 (13%)	1 (2.2%)	0	0
Donati et al., 2010 [20]	24 (77%)	7 (23%)	0	0	0	0	0
Donati et al., 2005 [21]	0	0	0	63 (100%)	0	0	0
Gitelis et al., 1981 [22]	·	·	·	·	·	·	·
Mavrogenis et al., 2013 [23]	0	0	0	119 (100%)	0	0	0
Mitchell et al., 2000 [10]	8 (57%)	2 (14%)	0	4 (29%)	0	0	0
Ozaki et al., 1996 [24]	6 (29%)	2 (9.5%)	0	13 (62%)	0	0	0
Staals et al., 2006 [25]	67 (54%)	27 (22%)	0	28 (23%)	0	0	0
van Maldegem et al., 2014 [26]	∧	∧	∧	63 (37%)	9 (5.3%)	15 (8.8%)	∧

^*∗*^If specifically mentioned;  ^∧^no differentiation possible.

**Table 3:** Oncologic outcome, survival.

Study	Overall survival	5 y survival	10 y survival	Grade 1 5 y survival	Grade 1 10 y survival
Andreou et al., 2011 [15]	63%	72%	69%	89%	89%
Angelini et al., 2012 [16]	84%	92%	84%	99%	95%
Briccoli et al., 2002 [17]	86%	·	·	·	·
Cho et al., 2011 [18]	84%	·	85%^∧^	·	·
de Camargo et al., 2010 [19]	94%^∧∧^	·	·	·	·
Donati et al., 2010 [20]	100%	100%	·	·	·
Donati et al., 2005 [21]	73%	·	·	·	·
Gitelis et al., 1981 [22]	·	49%	32%	·	·
Mavrogenis et al., 2013 [23]	·	80%^*∗*^	65%^*∗*^	·	·
Mitchell et al., 2000 [10]	21%	·	·	·	·
Ozaki et al., 1996 [24]	57%	·	·	82%	·
Staals et al., 2006 [25]	24%	24%	·	·	·
van Maldegem et al., 2014 [26]	·	2%	·	·	·

Study	Grade 2 5 y survival	Grade 2 10 y survival	Grade 3 5 y survival	Grade 3 10 y survival	Dedifferentiated 5 y survival

Andreou et al., 2011 [15]	63%	58%	39%	33%	·
Angelini et al., 2012 [16]	92%	86%	77%	55%	·
Briccoli et al., 2002 [17]	·	·	50%	·	·
Cho et al., 2011 [18]	·	85%^∧^	·	·	·
de Camargo et al., 2010 [19]	·	·	·	·	·
Donati et al., 2010 [20]	·	·	·	·	·
Donati et al., 2005 [21]	·	·	·	·	·
Gitelis et al., 1981 [22]	·	·	·	·	·
Mavrogenis et al., 2013 [23]	·	·	·	·	·
Mitchell et al., 2000 [10]	·	·	·	·	·
Ozaki et al., 1996 [24]	67%	·	0%	·	·
Staals et al., 2006 [25]	·	·	·	·	24%
van Maldegem et al., 2014 [26]	·	·	·	·	·

^*∗*^Extracted from Kaplan Meier curve, ^∧∧^43/46 = 93%, and ^∧^discrepancy calculation and manuscript.

**Table 4:** Oncologic outcome, survival.

Study	Disease-free survival	No evidence of disease	No evidence of disease after tumor relapse	Alive with disease	Dead of disease	Dead of other causes
Andreou et al., 2011 [15]	63%	73 (63%)	0	0	38^*∗*^ (33%)	4 (3.5%)
Angelini et al., 2012 [16]	79%	201 (68%)	33 (11%)	15 (5.1%)	35 (12%)	12 (4.1%)
Briccoli et al., 2002 [17]	71%	10 (71%)	·	2 (14%)	1 (7.1%)	1 (7.1%)
Cho et al., 2011 [18]	75%	24 (75%)	2 (6.3%)	3 (9.4%)	5 (16%)	0
de Camargo et al., 2010 [19]	89%	·	·	6 (13%)	3 (7%)	0
Donati et al., 2010 [20]	·	29 (94%)	2 (6.5%)	0	0	0
Donati et al., 2005 [21]	·	·	·	·	·	·
Gitelis et al., 1981 [22]	30%	·	·	·	·	·
Mavrogenis et al., 2013 [23]	·	·	·	·	·	·
Mitchell et al., 2000 [10]	·	·	·	·	·	·
Ozaki et al., 1996 [24]	62%	4 (19%)	9 (43%)	0	7 (33%)	1 (4.8%)
Staals et al., 2006 [25]	·	·	·	·	84 (76%)	·
van Maldegem et al., 2014 [26]	·	·	·	·	·	·

^*∗*^Including 6 treatment related deaths.

**Table 5:** 

Study	Local recurrence	Time to local recurrence (months)	Metastasis	Time to metastasis (months)	Chemotherapy	Radiation
Andreou et al., 2011 [15]	38 (33%)	21 (2–96)	30 (26%)	27 (2–141)	Used	Used
Angelini et al., 2012 [16]	50 (17%)	·	41 (14%)	·	Not used	Not used
Briccoli et al., 2002 [17]	6 (43%)	·	·	·	Used	·
Cho et al., 2011 [18]	2 (6.2%)	·	10 (31%)	49 (7–181)^*∗*^	·	·
de Camargo et al., 2010 [19]	16 (35%)	24 (9–46)	·	·	Not used	Not used
Donati et al., 2010 [20]	2 (6.5%)	31 (31–31)	0	·	·	·
Donati et al., 2005 [21]	15 (24%)	·	·	·	·	·
Gitelis et al., 1981 [22]	22 (32%)	·	26 (38%)	·	·	·
Mavrogenis et al., 2013 [23]	·	·	·	·	Used	·
Mitchell et al., 2000 [10]	·	·	·	·	Used	Used
Ozaki et al., 1996 [24]	·	·	·	·	Not used	Used
Staals et al., 2006 [25]	·	·	·	·	Used	.
van Maldegem et al., 2014 [26]	·	·	·	·	Used	Used

^*∗*^Different numbers calculable in paper.

**Table 6:** 

Study	Inadequate surgical margins	Wide and radical margin
	Enneking: intralesional or marginal	
Andreou et al., 2011 [15]	21 (18%)	94 (82%)
Angelini et al., 2012 [16]	74 (25%)	222 (75%)
Briccoli et al., 2002 [17]	3 (21%)	11 (79%)
Cho et al., 2011 [18]	7 (22%)	25 (78%)
de Camargo et al., 2010 [19]	25 (54%)	18 (39%)
Donati et al., 2010 [20]	17 (55%)	14 (45%)
Donati et al., 2005 [21]	17 (27%)	46 (73%)
Gitelis et al., 1981 [22]	37 (54%)	32 (46%)
Mavrogenis et al., 2013 [23]	.	.
Mitchell et al., 2000 [10]	.	.
Ozaki et al., 1996 [24]	21 (100%)	0
Staals et al., 2006 [25]	.	.
van Maldegem et al., 2014 [26]	52 (30%)^*∗*^	87 (51%)^*∗*^

^*∗*^Initial surgery.

**Table 7:** Prognostic factors.

Andreou et al. [15]	
Overall survival	

*Variable (bivariate analysis)*	*p value*
Sex	*p* = 0.6
Age (higher)	*p* = 0.04
Extremity versus axial + pelvis	*p* = 0.002
Tumor volume (0–100 cc vs. >100 cc)	*p* < 0.001
Grade tumor	*p* < 0.001
Local recurrences	*p* < 0.001
Distant metastasis	*p* < 0.001
Surgical margins	*p* = 0.9
Type of surgery	
Low grade: ablative versus limb-sparing	*p* = 0.7
High grade: ablative versus limb-sparing	*p* = 0.1
Pathologic fracture	*p* = 0.002
ACJCC	*p* < 0.001
Multi disc. versus support. care	*p* = 0.001
*Variable (multivariate analysis)*	
High grade: RR = 5	*p* < 0.001
Axial + pelvis: RR = 2	*p* = 0.04

Staals et al. [25]	
Disease-free survival	

*Variable (bivariate analysis)*	*p value*
Gender	NS
Age	NS
Duration of symptoms	NS
Lesion size	NS
Anatomic location	NS
Stage 3 versus Stage 2a	*p* = 0.003
Stage 3 versus Stage 2b	*p* < 0.00005
Stage 2a versus Stage 2b	*p* = 0.27
Histologic subtype, MFH versus OS	*p* = 0.046
Histologic subtype, MFH versus fibr. sarc.	*p* = 0.08
Histologic subtype, OS versus fibr. sarc.	*p* = 0.96
Grade 3DD versus grade 4DD	*p* = 0.10
Percentage of DD component	*p* = 0.0102
Percentage of DD component, >50% versus <50%	*p* < 0.00005
Limb-sparing versus resection	*p* = 0.08
Surgery versus surgery + chemotherapy	*p* = 0.88
*Variable (multivariate analysis, overall survival)*	
Percentage of DD component	*p* = 0.0102

Angelini et al. [16]	
Overall survival	

*Variable (bivariate analysis)*	*p value*
G1: wide versus intralesional	*p* = 0.495
G1: extremity versus trunk	*p* = 0.595
G2: wide versus intralesional	*p* = 0.948
G2: extremity versus trunk	*p* = 0.589
G2: resect. versus amputation	*p* = 0.496
G3: extremity versus trunk	*p* = 0.039
G3: resect. versus amputation	*p* = 0.051
*Variable (multivariate analysis)*	
G3: resect. versus amputation	*p* = 0.0943
G3: extr. versus trunk	*p* = 0.0889

van Maldegem et al. [26]	
Overall survival from the day of unresectability	

*Variable (bivariate analysis)*	*p value*
Only local unresectable disease versus local unresectable disease + metastasis	*p* = 0.0014
Age (<40 years)	*p* = 0.001
Grade II tumors	*p* = 0.022
Sex	NS
Site	NS
Resectable versus nonresectable disease at primary diagnosis	NS
Systemic treatment	*p* < 0.0487

RR = Relative Risk, G = grade, MFH = malignant fibrohistocytoma, OS = osteosarcoma, and DD = dedifferentiated.

**(a):** 

Author, year	Study participation: Dates of researched period stated Clearly defined patient sample, assembled at a common point in course of the disease	Study attrition: Sufficiently long and complete follow-up (≥2 years and ≥80%) Explaining reasons for patients being lost to follow-up	Confounding measurement: Defined and comparable treatment for patients
Andreou et al., 2011 [15]	1	1	1, types of surgery mentioned
Angelini et al., 2012 [16]	1	1	1, types of surgery mentioned
Briccoli et al., 2002 [17]	1	0	1, types of surgery mentioned
Cho et al., 2011 [18]	1	1	1, types of surgery mentioned
de Camargo et al., 2010 [19]	1	1	1, types of surgery mentioned
Donati et al., 2010 [20]	1	1	1, types of surgery mentioned
Donati et al., 2005 [21]	1	1	1, types of surgery mentioned
Gitelis et al., 1981 [22]	1	1	1, types of surgery mentioned
Mavrogenis et al., 2013 [23]	1	1	1, types of surgery mentioned
Mitchell et al., 2000 [10]	1	0	1, types of surgery mentioned
Ozaki et al., 1996 [24]	1	1	1, types of surgery mentioned
Staals et al., 2006 [25]	1	0	1, types of surgery mentioned
van Maldegem et al., 2014 [26]	1	0	1, types of surgery mentioned

**(b):** 

Author, year	Analysis: Valid statistical analysis is done Multivariable analysis is done	Population: (no overlap)	Disclosure
Andreou et al., 2011 [15]	1	1	1
Angelini et al., 2012 [16]	1	0	1
Briccoli et al., 2002 [17]	0	0	0
Cho et al., 2011 [18]	0	1	1
de Camargo et al., 2010 [19]	0	1	1
Donati et al., 2010 [20]	0	0	1
Donati et al., 2005 [21]	1, but not on central survival	0	1
Gitelis et al., 1981 [22]	0	0	0
Mavrogenis et al., 2013 [23]	1	0	1
Mitchell et al., 2000 [10]	0	1	1
Ozaki et al., 1996 [24]	0	1	0
Staals et al., 2006 [25]	0	0	0
van Maldegem et al., 2014 [26]	0	0	1

**(c):** 

Author, year	Prognostic factor measurement: Clear definition and valid assessment of prognostic factors	Outcome measurement: Well defined outcome parameters (survival: overall, metastatic-free, event-free)

Andreou et al., 2011 [15]	1	1
Angelini et al., 2012 [16]	1	1
Briccoli et al., 2002 [17]	0	0
Cho et al., 2011 [18]	1	1
de Camargo et al., 2010 [19]	1	1
Donati et al., 2010 [20]	1	0
Donati et al., 2005 [21]	1	0
Gitelis et al., 1981 [22]	1	1
Mavrogenis et al., 2013 [23]	1	1
Mitchell et al., 2000 [10]	1	0
Ozaki et al., 1996 [24]	1	1
Staals et al., 2006 [25]	1	1
van Maldegem et al., 2014 [26]	1	1

**(d):** 

Author, year	FU >1 year	Level of evidence I–IV
Andreou et al., 2011 [15]	1	4, prognostic
Angelini et al., 2012 [16]	1	4, prognostic
Briccoli et al., 2002 [17]	0	4, prognostic
Cho et al., 2011 [18]	1	3, therapeutic
de Camargo et al., 2010 [19]	1	4, prognostic
Donati et al., 2010 [20]	1	2, prognostic
Donati et al., 2005 [21]	1	4, prognostic
Gitelis et al., 1981 [22]	1	4, prognostic
Mavrogenis et al., 2013 [23]	1	4, prognostic
Mitchell et al., 2000 [10]	1	4, prognostic
Ozaki et al., 1996 [24]	1	4, prognostic
Staals et al., 2006 [25]	0	4, prognostic
van Maldegem et al., 2014 [26]	1	4, prognostic

**(e):** 

Author, year	Confounding measurement Defined and comparable treatment for patients	Baseline

Andreou et al., 2011 [15]	1, types of surgery mentioned	0
Angelini et al., 2012 [16]	1, types of surgery mentioned	0
Briccoli et al., 2002 [17]	1, types of surgery mentioned	1
Cho et al., 2011 [18]	1, types of surgery mentioned	0
de Camargo et al., 2010 [19]	1, types of surgery mentioned	0
Donati et al., 2010 [20]	1, types of surgery mentioned	1
Donati et al., 2005 [21]	1, types of surgery mentioned	0
Gitelis et al., 1981 [22]	1, types of surgery mentioned	1
Mavrogenis et al., 2013 [23]	1, types of surgery mentioned	0
Mitchell et al., 2000 [10]	1, types of surgery mentioned	1
Ozaki et al., 1996 [24]	1, types of surgery mentioned	1
Staals et al., 2006 [25]	1, types of surgery mentioned	0
van Maldegem et al., 2014 [26]	1, types of surgery mentioned	1

**(f):** 

Author, year	Disclosure

Andreou et al., 2011 [15]	1
Angelini et al., 2012 [16]	1
Briccoli et al., 2002 [17]	0
Cho et al., 2011 [18]	1
de Camargo et al., 2010 [19]	1
Donati et al., 2010 [20]	1
Donati et al., 2005 [21]	1
Gitelis et al., 1981 [22]	0
Mavrogenis et al., 2013 [23]	1
Mitchell et al., 2000 [10]	1
Ozaki et al., 1996 [24]	0
Staals et al., 2006 [25]	0
van Maldegem et al., 2014 [26]	1

## Appendix

See Table 8.
